# Antibacterial Activity and Transcriptomic Analysis of Hesperetin against *Alicyclobacillus acidoterrestris* Vegetative Cells

**DOI:** 10.3390/foods12173276

**Published:** 2023-09-01

**Authors:** Siqi Zhao, Yanzi Nan, Runyu Yao, Langhong Wang, Xinan Zeng, Rana Muhammad Aadil, Muhammad Asim Shabbir

**Affiliations:** 1 School of Food Science and Engineering, South China University of Technology, Guangzhou 510641, China; 202120126516@mail.scut.edu.cn (S.Z.); 202320126387@mail.scut.edu.cn (Y.N.); 202110185808@mail.scut.edu.cn (R.Y.); wlhong@fosu.edu.cn (L.W.); 2 School of Food Science and Engineering, Guangdong Provincial Key Laboratory of Intelligent Food Manufacturing, Foshan University, Foshan 528225, China; 3 National Institute of Food Science and Technology, University of Agriculture, Faisalabad 38000, Pakistan; muhammad.aadil@uaf.edu.pk (R.M.A.); asim-shabbir@live.com (M.A.S.)

**Keywords:** *Alicyclobacillus acidoterrestris*, hesperetin, antibacterial activity, antibacterial mechanism, transcriptomic analysis

## Abstract

The aim of this research was to investigate the antimicrobial characteristics and mechanism of hesperetin against *Alicyclobacillus acidoterrestris* vegetative cells. The results presented show that hesperetin had effective antimicrobial activity on *Alicyclobacillus acidoterrestris* vegetative cells, minimum inhibition concentration (MIC) of 0.0625 g/L, and minimum bacterial concentration (MBC) greater than 2 g/L. Moreover, treatment of hesperetin caused significant damage to cell integrity, preventing the growth of *Alicyclobacillus acidoterrestris* vegetative cells, enhancing the leakage of nucleic acid and proteins, and destroying the vegetative cell morphology. To further investigate the mechanism, transcriptomic analysis was carried out, and 3056 differentially expressed genes (DEGs) were detected. Gene ontology (GO) enrichment analysis revealed that hesperetin inhibits *Alicyclobacillus acidoterrestris* by affecting the intracellular nitrogen metabolism and amino acid metabolism. The Kyoto Encyclopedia of Genes and Genomes (KEGG) enrichment analysis explained that hesperetin was also able to prevent the growth of *Alicyclobacillus acidoterrestris* by affecting the processes of nutrient transport, energy metabolism, and flagella motility. These results provide new insights into the antimicrobial effects and mechanism of hesperetin against *Alicyclobacillus acidoterrestris*, which provides a new method for inactive *Alicyclobacillus acidoterrestris* in the juice industry.

## 1. Introduction

*Alicyclobacillus acidoterrestris* is a spore-forming, non-pathogenic Gram-positive bacterium with good thermophilic and acidophilic properties. It can produce guaiacol (2-methoxyphenol) and halophenol (2,6-dibromophenol), which can cause spoilage in fruit juice drinks [1]. The sensory threshold level of guaiacol is very low, while the threshold level for smelling guaiacol in water (0.02 mg/L), 12% aqueous ethanol (0.03 mg/L), and dry white wine are (0.02 mg/L) [2]. This bacterium is difficult to find in the early stage of growth. When the external environment is not suitable for growth, *A. acidoterrestris* can produce spores. Once the environment is suitable, it can rapidly grow and reproduce; then, it starts to pollute the juice and produce a foul odor. Moreover, its spores have strong resistance and can survive under extreme conditions [3]. Due to this problem, *A. acidoterrestris* has attracted more and more attention from juice producers. In recent years, non-thermal techniques have been used to control and inactivate *A. acidoterrestris* vegetative cells and spores, such as UV sterilization [4], ultrasonic treatment [5], ohmic heating [6], cold plasma [7], and natural compounds [8]. As one of the natural compounds, flavonoids are heterocyclic organic compounds that have been proven to have antibacterial, antioxidant, and anti-inflammatory biological activities, which are widely found in fruits and vegetables [9]. The basic nucleus of flavonoids is 2-phenylchromone, and two benzene rings (A ring and B ring) are connected in the way of a C_6_-C_3_-C_6_ manner. It has been reported that the antibacterial mechanism of flavonoids is mainly divided into two aspects: (1) the permeability of the bacterial cell membrane is altered, which can cause the outflow of the substances in the cytoplasm. (2) The synthesis of bacterial nucleic acids is inhibited (inhibitory activity varies with substituents) [10].

Hesperetin (5,7,30-trihydroxy-40-methoxyflavanone) is a flavonoid found in citrus fruits of the Rutaceae family, usually in the form of its glycoside and hesperidin, which is present in the peel of fruit [11]. In general, Citrus bioflavonoids, including hesperetin and its glycoside form, appear to be extremely safe and without side effects [12,13]. At present, a large number of studies have been conducted to show that hesperetin has a variety of biological activities such as antioxidant [14], anti-inflammatory [15], and antibacterial [16,17,18]. At the same time, hesperetin, as a kind of natural active substance with antibacterial ability, has a great impact on the antibacterial aspect of food processing. Liang et al. isolated hesperetin from orange peel and carried out antibacterial experiments. Their results showed that hesperetin showed broad-spectrum antibacterial activity against *Bacillus subtilis*, *Staphylococcus aureus*, and Methicillin-resistant *Staphylococcus aureus* (MRSA) [17]. As per our knowledge, no research has been reported on the antimicrobial activity and mechanism of *A. acidoterrestris* treated by hesperetin.

In this work, the inhibitory effect of hesperetin on the cell growth of *A. acidoterrestris* was detected. The leakage of nucleic acid, proteins, and morphological changes were analyzed. To further gain insights into the possible antimicrobial molecular mechanisms of hesperetin against *A. acidoterrestris* vegetative cells, transcriptomics was also investigated. The findings in this work are expected to provide a broader understanding of hesperetin as a natural antibacterial substance to control *A. acidoterrestris* but also hold promising implications for the development of novel strategies to prevent *A. acidoterrestris* contamination in the fruit beverage industry.

## 2. Materials and Methods

### 2.1. Raw Materials and Chemicals

Not from concentrate (NFC) apple juice was purchased from Beijing Top Flier Import & Export Co., Ltd. (Beijing, China). Hesperetin (>99.0%) was provided by Shanghai Aladdin Biochemical Technology Co., Ltd. (Shanghai, China). PI and SYTO 9 dyes were obtained from Thermo Fisher Scientific Inc. (Shanghai, China).

### 2.2. Bacterial Strain and Cultures

*A. acidoterrestris* (ATCC 49025) was obtained from the Guangdong Microbial Culture Collection Center (Guangzhou, China). The strain was subcultured in *A. acidoterrestris* medium (AAM, D-(+)-glucose (99%)) 0.5 g, potassium phosphate monobasic (KH_2_PO_4_, ≥99.0%) 0.3 g, magnesium sulfate (MgSO_4_·7H_2_O, ≥99.0%) 0.25 g, ammonium sulfate ((NH_4_)_2_SO_4_, ≥99.0%) 0.2 g, yeast 0.5 g, and calcium chloride dihydrate (CaCl_2_·2H_2_O, ≥99.0%) 0.095 g and 200 mL deionized water) at 45 °C for 16–20 h.

### 2.3. Antimicrobial Activity Tests

#### 2.3.1. Minimum Inhibitory Concentration (MIC) and Minimum Bacterial Concentration (MBC)

The minimum inhibitory concentration (MIC) and minimum bacterial concentration (MBC) were determined by a ten-fold dilution method. All test tubes were incubated in a 45 °C incubator for 24 h. The MIC was the lowest concentration in all test tubes where turbidity is not visible to the naked eye. The subcultured samples were plated on AAM agar medium and incubated at 45 °C for 48 h for observation. The lowest concentration at which no bacterial growth was visible to the naked eye in all plates was the MBC [19].

#### 2.3.2. Growth Curves and Kinetic Parameters

Hesperetin was added to the *A. acidoterrestris* suspension (10^7^–10^8^ CFU/mL), and *A. acidoterrestris* without hesperetin was used as a negative control. The bacterial suspensions were incubated for 24 h at 45 °C, during which the samples were collected every 2 h and tested for absorbance at 600 nm using a NanoDrop spectrophotometer (ND-2000, Saveen Werner, USA). The growth curve and Gompertz curve were obtained by fitting the modified Gompertz equation (Equation (1)) to the data [20].

(1) ODt=A+(B − A) × exp [−exp (−r × (t − M))],

where ODt is the optical density of bacterial samples at time t (h), A is the initial optical density, B is the maximum optical density, M is the time (h) at which the absolute growth rate is maximum, and r is the relative growth rate at M. The growth parameters, including lag phase duration λ (λ = M − (1/r)), maximum growth rate μ (μ = (B − A) × r/e), and generation time Tg (Tg = log [2 × e*/*(r × (B − A))]), were calculated [21].

### 2.4. Determination of Morphophysiological Properties

#### 2.4.1. Fluorescence Microscopy

The *A. acidoterrestris* was incubated with increasing concentrations of hesperetin (1/2 MIC and MIC) at 45 °C for 2 h. The treated and control samples were washed with PBS buffer three times and then stained with PI and SYTO 9 dyes for 15 min under dark conditions. After that, the *A. acidoterrestris* vegetative cells were centrifuged to remove excess dyes and resuspend the cells in PBS buffer. Each obtained sample (5 μL) was placed on a glass slide, and images were captured using a fluorescence microscope (Leica DMi8, Wetzlar, Germany) [22].

#### 2.4.2. Scanning Electron Microscopy (SEM)

The effect of hesperetin on the ultrastructural changes in *A. acidoterrestris* vegetative cells was observed by SEM. The *A. acidoterrestris* vegetative cells were first treated with hesperetin (1 × MIC and 2 × MIC) at 45 °C for 2 h and then centrifuged at 4000× *g* for 10 min at 4 °C, then wash the samples thrice with PBS buffer. The treated and control *A. acidoterrestris* vegetative cells were fixed with 2.5% glutaraldehyde for 12 h at 4 °C. After the fixation process, the samples were subjected to gradient dehydration for 15 min each, with ethanol solution at a concentration of 30, 50, 70, 80, 90, and 100%, respectively. Finally, the *A. acidoterrestris* vegetative cells were dried and coated with gold. The images were observed by scanning electron microscope (S-4800, Hitachi, Tokyo, Japan) [23].

### 2.5. Leakage of DNA, RNA, and Proteins

The permeability change in the *A. acidoterrestris* vegetative cell membrane treated with hesperetin was studied according to the release of intracellular DNA, RNA, and proteins. Bacterial suspensions (10^7^ CFU/mL) were treated with hesperetin (1 MIC and 2 MIC) and then incubated at 45 °C for 3, 6, and 24 h, respectively. The leakage of nucleic acids and proteins was measured using a NanoDrop spectrophotometer (ND-2000, Saveen Werner, USA) to record the absorbance at 260 and 595 nm, respectively [1].

### 2.6. RNA Extraction

The *A. acidoterrestris* vegetative cells were first treated with hesperetin (1 × MIC and 2 × MIC) at 45 °C for 2 h. The hesperetin-treated cells were resuspended in an EP tube containing lysozyme and TE buffer, mixed well, and incubated at 25 °C for 5 min. RNA extraction, cDNA library preparation, and RNA sequencing were conducted following the previous study of Song et al. [24]. Following the manufacturer’s instructions, the total RNA was extracted from each sample using TRIzol reagent (Invitrogen, Carlsbad, CA, USA); after extraction, the RNA samples were treated with RNase-free DNase I (ThermoFisher Scientific, Waltham, MA, USA) to determine the quality of RNA samples using 1% agarose gel electrophoresis. The quantity of RNA samples was measured by a NanoDrop Spectrophotometer (ND-2000, Saveen Werner, USA). The qualified RNA was sent to the Beijing Genomics Institute (BGI), Shenzhen, for transcriptomic sequencing using an Illumina X-TEN platform. After being treated with RNase-free DNase I, magnetic beads with Oligo (dT) were used to enrich the mRNA. The cDNA was synthesized using the mRNA fragments as templates.

### 2.7. Transcriptomic Data Processing

The sequencing data were filtered with the software SOAPnuke (v1.5.6) by (1) removing reads containing a sequencing adapter, (2) removing reads whose low-quality base ratio (base quality less than or equal to 15) was more than 20%, and (3) removing reads whose unknown base (‘N’ base) ratio was more than 5%. The expression level of the *A. acidoterrestris* cells gene was calculated by RSEM software (v1.3.1). The heatmap was drawn by heatmap (v1.0.8) according to the gene expression difference in different samples. The gene expression level of untreated *A. acidoterrestris* vegetative cells at 4 °C was regarded as a reference; whether a gene from the treated cells was upregulated or downregulated was determined by comparing its expression level with that at 4 °C. Essentially, differential expression genes (DEGs) analysis was performed using the DESeq2 (v1.4.5) software; fold change ≥2 and adjusted *p*-value ≤ 0.001 were the standard for screening genes with significant differential expression.

According to gene ontology (GO) and the Kyoto Encyclopedia of Genes and Genomes (KEGG) annotation results and classifications, the differentially expressed genes were functionally classified, and the Phyper function in R software was used for KEGG enrichment analysis. With a Q value of ≤0.05 as the threshold, candidate genes that meet this condition were defined as significantly enriched.

### 2.8. Statistical Analyses

All the treatments were performed in triplicate, and the experimental data were presented as the mean ± standard deviation (SD). The related data were analyzed by variance (ANOVA) followed by the Tukey test using SPSS 22.0 software (IBM, New York, NY, USA) and GraphPad Prism 8 software (San Diego, CA, USA), and *p*-values < 0.05 were regarded as significantly different (* *p* ≤ 0.05, ** *p* ≤ 0.01, *** *p* ≤ 0.001, and **** *p* ≤ 0.0001).

## 3. Results

### 3.1. Antimicrobial Activity of Hesperetin on A. acidoterrestris

#### 3.1.1. MIC and MBC of Hesperetin against *A. acidoterrestris*

**Figure 1:**
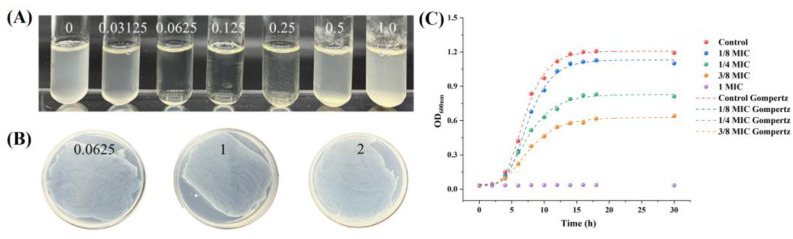
Antibacterial activity of hesperetin on *A. acidoterrestris* vegetative cells: MIC (**A**), MBC (**B**), and growth curves (**C**).

The MIC and MBC of hesperetin against *A. acidoterrestris* were determined and are shown in Figure 1. From Figure 1A, it can be found that the concentration regarding the first clear test tube from left to right was 0.0625 g/L, so the MIC of hesperetin against *A. acidoterrestris* vegetative cells was 0.0625 g/L. The results revealed that hesperetin possessed good antibacterial activity against *A. acidoterrestris*. It was also found that when the concentration of hesperetin reached 0.5 g/L, the tube became turbid. This is because hesperetin was poorly dissolved in the bacterial suspension equal to or higher than this concentration. Figure 1B revealed that the MBC of hesperetin against *A. acidoterrestris* vegetative cells was more than 2 g/L. This finding implied that *A. acidoterrestris* might possess potential adaptive mechanisms that enable it to develop tolerance to hesperetin even at relatively low concentrations.

#### 3.1.2. Effect of Hesperetin on *A. acidoterrestris* Growth

The growth curve was determined, as shown in Figure 1C, based on the MIC value. *A. acidoterrestris* was totally inhibited when hesperetin was at the optimal MIC concentration. Compared to the control sample, *A. acidoterrestris* can grow at concentrations lower than MIC. Gompertz curves were also generated for cultivation at four different conditions (1/8 MIC, 1/4 MIC, 3/8 MIC, and control) by fitting the modified Gompertz model to the data. Meanwhile, the growth kinetic parameters of *A. acidoterrestris* were calculated according to the fitting results (Table 1). As expected, with increasing hesperetin concentration, μ (the maximum growth rate) and OD_max_ (the maximum population density) were significantly lower than the control sample (*p* < 0.05), while λ (the lag phase duration) and Tg (the generation time) were significantly increased (*p* < 0.05). The trend of Gompertz curves treated by hesperetin was similar to the previous study that used other natural compounds on *A. acidoterrestris* [20,21].

### 3.2. Effect of Hesperetin on A. acidoterrestris Morphology

#### 3.2.1. Fluorescent-Based Cell Live/Dead Test

**Figure 2:**
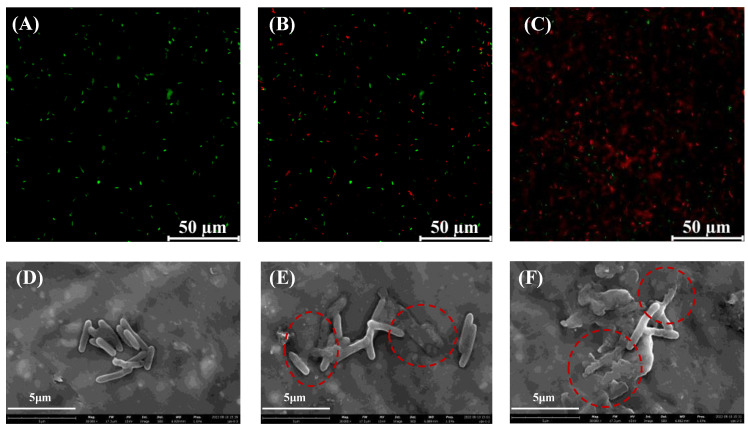
Fluorescence microscope and scanning electron microscopic (SEM) photomicrographs of *A. acidoterrestris* vegetative cells treated with hesperetin at 0 MIC (**A**,**D** control), (**B**,**E**) 1 MIC, and (**C**,**F**) 2 MIC. Note: the red circle indicates that the cell membrane has been damaged.

To elucidate the role of *A. acidoterrestris* vegetative cell membrane damage by hesperetin, the damage of the *A. acidoterrestris* cell membrane was investigated via the fluorescence-based dye test. Fluorescence images are presented in Figure 2A–C. Both live and dead cells of *A. acidoterrestris* exhibited similar green fluorescence after SYTO9-staining alone, whereas dead cells of *A. acidoterrestris* showed red fluorescence when counterstained with propidium iodide (PI). PI can enter *A. acidoterrestris* cells with damaged membranes and bind to DNA or RNA to produce red fluorescence[17]. As shown in Figure 2A, the control cells possess green fluorescence without red fluorescence. In contrast, 1/2 MIC hesperetin-treated *A. acidoterrestris* cells show both green and red fluorescence, revealing that hesperetin has the ability to inactivate *A. acidoterrestris* vegetative cells. Moreover, it can be clearly observed that the large majority of *A. acidoterrestris* cells present red fluorescence after being treated with 1 MIC hesperetin, which demonstrates that the loss of *A. acidoterrestris* cell viability is positively correlated with the concentration of hesperetin, and it can be concluded that hesperetin treatment leads to the loss of *A. acidoterrestris* cell integrity. Jia et al. utilized the same fluorescent staining microscopy method to verify the mechanism of Epsilon-Polylysine-Based Magnetic Nanoflowers treated with *A. acidoterrestris*. In their research, it was found that the loss of cell viability gradually increased with the concentration of nanocomposites [25].

#### 3.2.2. SEM Observation of Hesperetin-Treated Cells

To further determine the inactivation mechanism of hesperetin-treated *A. acidoterrestris* vegetative cells, SEM was performed. As depicted in Figure 2D, it can be seen that untreated *A. acidoterrestris* vegetative cells displayed an intact and smooth cell surface. As the concentration of hesperetin increased by 1/2 MIC, the bacterial morphology had a slight effect (Figure 2E). *A. acidoterrestris* cells treated with hesperetin at the MIC level were atrophied and irregular and exhibited some degree of collapse on the surface (Figure 2F). The result revealed that hesperetin had a destructive effect on *A. acidoterrestris* vegetative cells and could lose the viability of *A. acidoterrestris* cells. Cai et al. reported that after 1 MIC thymol was treated, *A. acidoterrestris* cells became irregular and showed hollowness on the cell surface [19]. Previous studies indicated that the natural compound could penetrate the bacterial cells and trigger an increase in cell membrane permeability, leading to the loss of the normal physiological function of *A. acidoterrestris* and resulting in cell death [26,27].

### 3.3. Leakage of Biomolecules

**Figure 3:**
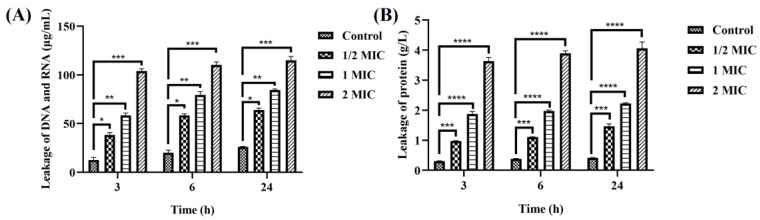
Nucleic acid (**A**) and proteins (**B**) leakage from *A. acidoterrestris* vegetative cells treated with hesperetin for 3, 6, and 24 h. Data represent means ± SD of three independent experiments with duplicate measurements of cell counts. Different symbols indicate that the corresponding values are significantly different (Tukey test, * *p* ≤ 0.05, ** *p* ≤ 0.01, *** *p* ≤ 0.001, and **** *p* ≤ 0.0001).

To demonstrate the effect of hesperetin treatment on *A. acidoterrestris* vegetative cells, the leakage of intracellular content was determined. Figure 3A,B shows the leakage of nucleic acids and protein in *A. acidoterrestris* vegetative cells under different hesperetin concentrations, respectively. The leakage of nucleic acid increased with the increasing hesperetin concentration. In Figure 3A, the leakage of DNA and RNA in the control bacterial sample at 3, 6, and 24 h were 12.5, 20.12, and 26.03 μg/mL, respectively. After treating the 1/2 MIC hesperetin sample, the corresponding values were 38.26, 58.37, and 63.92 μg/mL. When the concentration reached 1 MIC, the leakage amounts were 59.48, 79.6, and 84.46 μg/mL. Moreover, when treated with hesperetin at the 2 MIC level for 3 h, the leakage of DNA and RNA was 97.39 μg/mL, but there were no significant changes when the exposure time was increased to 6 and 24 h. The same trend was reported in previous studies [1,19].

The leakage of proteins from *A. acidoterrestris* was increased with the hesperetin concentration, as shown in Figure 3B. There was a significant difference between the control sample and the hesperetin-treated samples. When the exposure time was 3 h, the leakage of protein from the control sample was 0.3 g/L, and the corresponding values of 1/2 MIC, 1 MIC, and 2 MIC were 3.23, 12.4, and 24.27 g/L times higher, respectively. In addition, prolonged treatment time had little effect on the leakage of proteins. These results indicate that hesperetin treatment led to the lysis of biomolecules such as DNA, RNA, and proteins, which then resulted in cell death.

### 3.4. Transcriptomic Effects of Hesperetin on A. acidoterrestris

#### 3.4.1. Differentially Expressed Genes (DEGs)

The preceding investigations demonstrated that hesperetin could severely disrupt the cell membrane of *A. acidoterrestris* and produce biomolecule leakage. Transcriptomic analysis was performed to further investigate the mode of action of hesperetin against *A. acidoterrestris* vegetative cells. The *A. acidoterrestris* cultured to the stable stage were collected and divided into 1/2 MIC and 1 MIC hesperetin treatment groups and a control group, each with three parallels. It can be seen in Table 2 that among the nine data samples, the Q20 of all samples was higher than 96.79%, the Q30 of all samples was higher than 91.91%, and the sequence alignment between each group of samples and the reference genome was high (≥95.64%). All sample data had high reliability and could participate in subsequent analysis.

**Figure 4:**
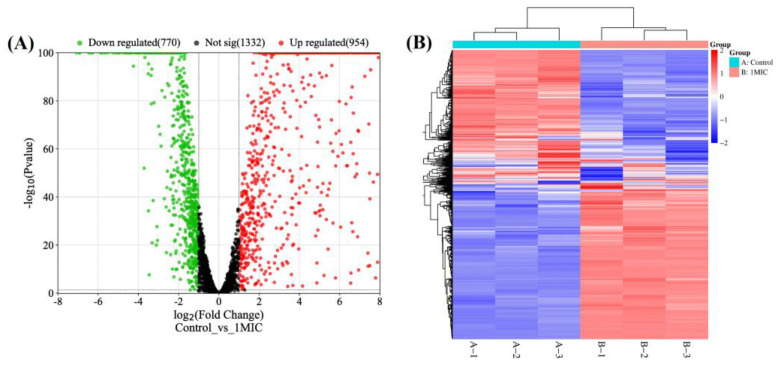
Volcano map (**A**) and cluster map (**B**) of *A. acidoterrestris* differential genes from gene list control_vs_1 MIC.

The control and hesperetin-treated samples were clustered separately, as depicted in Figure 4. Cluster analysis is usually used to measure the similarity of expression between samples. Hierarchical cluster analysis was performed based on the expression level of FPKM of differential genes. The cluster analysis results are shown in Figure 4A. Red indicates high gene expression, and blue indicates low gene expression. Samples with similar expression patterns were clustered together. The abscissa represents the clustering of samples, and each column represents one sample. The more similar the gene expression in the samples, the closer they will be. The vertical axis represents gene clustering; each row represents a gene, and the more similar the expression, the closer it is. It can be seen from the overall clustering results that different treatment conditions caused the upregulation and downregulation of some genes involved in the same biological process, indicating that some metabolic processes or cellular pathways play a role in the process of cells being treated with hesperetin [28]. The volcano plots of the hesperetin-treated and control groups are depicted in Figure 4A. It can be seen intuitively from the figure that compared with the control group, the number of the DEGs was 3056 (expression fold change ≥ 2 and *p*-value < 0.5). The number of genes upregulated and downregulated in the 1 MIC hesperetin treatment group were 954 and 770, respectively, and there were 1332 genes with a non-significant difference.

#### 3.4.2. GO and KEGG Annotation Analysis of DEGs

**Figure 5:**
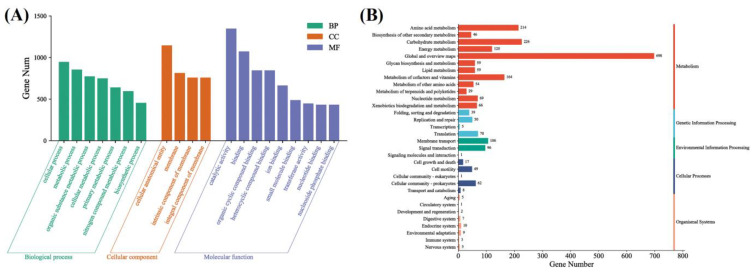
GO (**A**) and KEGG (**B**) annotation of *A. acidoterrestris* gene list control_vs_1 MIC.

The gene ontology (GO) database (http://www.geneontology.org/) is mainly divided into three functional categories: molecular functions, cellular components, and biological processes. GO functional classification was performed on the gene set, and the results are shown in Figure 5A. It can be seen from Figure 5A that the main biological processes involved are the cell process, metabolic process, organic substance metabolic process, and so on. For the cellular component category, the majority of DEGs were associated with a cellular anatomical entity, a membrane, an intrinsic component of the membrane, and an integral component of the membrane. In terms of molecular function, the most significantly enriched DEGs belonged to catalytic activity, binding, organic cyclic compound binding, ion binding, and so on. Using the KEGG database, all DEGs in the gene set were annotated and analyzed, and the pathway classification diagram is presented in Figure 5B. The majority of DEGs were associated with amino acid metabolism, carbohydrate metabolism, metabolism of cofactors and vitamins, energy metabolism, membrane transport, signal transduction, and so on.

#### 3.4.3. Functional Enrichment of GO and KEGG

**Figure 6:**
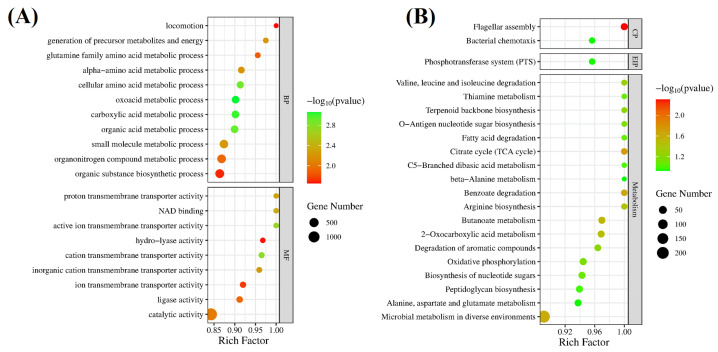
GO (**A**) and KEGG (**B**) enrichment analysis of *A. acidoterrestris* gene list control_vs_1 MIC.

According to the *p*-value, the Top 20 significant GO terms are depicted in Figure 6A. It can be seen from the figure that all genes were significantly enriched in GO terms such as catalytic activity, organic substance biosynthetic process, organonitrogen compound metabolic process, and small molecule metabolic process, and the number of genes was 1348, 431, 402, and 374, respectively. The results of GO enrichment analysis showed that after hesperetin treatment, the gene transcription level of *A. acidoterrestris* cell membrane production and metabolism increased, and the content of membrane components increased. In addition, hesperetin also affected the intracellular nitrogen metabolism and amino acid metabolism of *A. acidoterrestris*.

All DEGs in the gene set were enriched into 191 KEGG gene pathways, out of which the 20 most significant gene pathways are shown in Figure 6B. It can be found that all genes are significantly enriched in KEGG pathways such as flagellar assembly, citrate cycle (TCA cycle), benzoate degradation, arginine biosynthesis, and valine, leucine, and isoleucine degradation, and the number of genes is 32, 24, 23, 20, and 18, respectively. The results of KEGG enrichment analysis showed that hesperetin might play an antibacterial role by regulating *A. Acidoterrestris* amino acid metabolism, nutrient transport, energy metabolism, and other pathways.

## 4. Discussion

Hesperetin is one of the natural compounds of flavonoids in citrus fruits with good potential bacterial activity against several bacterial, such as *E. coli* (MIC_90_, 244.4 μg/mL) [10] and against *B. subtilis*, *S. aureus*, *Xanthomonas citri* subsp. *citri*, and meticillin-resistant *S. aureus* with MIC values of 0.0625, 0.0625, 0.0625, and 0.125 mg/mL, respectively [17]. Additionally, the MBCs were 0.5, 4.0, 0.2, and >1.0 mg/mL. Previously, as for *A. acidoterrestris*, it was reported that the MICs of cinnamic acid, chlorogenic [19], *p*-coumaric acid [20], and curcumin [29] were 0.375, 2.0, 0.2, and 1.0 mg/mL, respectively. These results suggest that hesperetin exhibited stronger antibacterial activity towards *A. acidoterrestris* vegetative cells, providing a highly effective option for controlling and preventing *A. acidoterrestris* vegetative cells, resulting in juice contamination.

Flavonoids, including naringenin and kaempferol, have been noted for their potent antibacterial properties attributed to their interaction with lipid bilayers, causing disruptions, alterations in membrane fluidity, and membrane damage [30]. A previous study demonstrated that the flavonoid compound 2R, 3R-dihydromyricetin induces disorder in membrane lipid arrangement, resulting in a significant modification of membrane fluidity in *S. aureus* cells [31]. Similarly, chrysin and quercetin exhibited analogous behavior [32]. Given its structural resemblance, hesperetin displays a parallel configuration, with hydroxyl substitutions at the 5’ and 7’ positions of the A ring and a methoxy substitution at the 3’ position of the C ring. This structural similarity may potentially explain the obvious antibacterial activity of hesperetin.

It is worth noting that the growth of biofilm is inseparable from the movement of bacteria. Bacterial flagella mediate the movement of bacteria [33]. The flagella structure is mainly composed of a basal body, hook, and filament. The dynamical system of flagellar movement is also called the flagellar motor, which is the most complex part of the flagellar and is responsible for the assembly, rotation, and direction-switching of the flagellar [34]. The flagellar motor consists of a stator and a rotor. The rotor contains several stacked ring-chain structures, in which the C ring is mainly composed of FliM, FliG, and FliN, and the MS ring is composed of the protein FliF [35]. After hesperetin treatment, more than ten flagellar motility-related genes were significantly downregulated in *A. acidoterrestris* (Table 3).

## 5. Conclusions

In conclusion, this research reported that hesperetin possessed effective antibacterial activity against *A. acidoterrestris*, with the MIC being 0.0625 g/L and MBC > 2g/L. The addition of hesperetin destroyed the integrity of *A. acidoterrestris* cells by inhibiting the growth process, enhancing the leakage of biomolecules (DNA, RNA, and proteins), and causing irreversible damage to bacterial morphology. The antimicrobial mechanism was characterized by transcriptomic analysis; hesperetin was also able to prevent the growth of *A. acidoterrestris* by affecting the processes of nutrient transport, energy metabolism, and flagella motility. As far as we know, these findings are the first report on the antibacterial activity and mechanism of hesperetin against *A. acidoterrestris* and lay a theoretical foundation for the prevention of *A. acidoterrestris* contamination in the beverage industry.

## Figures and Tables

**Table 1:** Kinetic parameters of *A. acidoterrestris* during growth with hesperetin at different concentrations.

Hesperetin	Growth Parameters				Gompertz Equations	R^2^
	λ	μ	OD_max_	Tg		
0 MIC (Control)	3.7965	0.1868	1.193	1.0296	OD_t_ = 0.0379 + 1.169 ∗ exp [−exp (−0.4329 ∗ (t − 6.1063))]	0.9980
1/8 MIC	4.1608	0.1647	1.101	1.0843	OD_t_ = 0.0424 + 1.0882 ∗ exp [−exp (−0.4102 ∗ (t − 6.5987))]	0.9985
1/4 MIC	4.4206	0.1025	0.811	1.2905	OD_t_ = 0.0269 + 0.8013 ∗ exp [−exp (−0.3465 ∗ (t − 6.1263))]	0.9974
3/8 MIC	5.2948	0.0718	0.64	1.4448	OD_t_ = 0.0294 + 0.5972 ∗ exp [−exp (−0.3259 ∗ (t − 6.3629))]	0.9983

**Table 2:** The data statistics of transcriptome sequencing.

Sample	Total Raw Reads (M)	Total Clean Reads (M)	Clean Reads Q20 (%)	Clean Reads Q30 (%)	Total Mapping (%)
Control-1	62.47	61.81	96.89	92.14	95.84
Control-2	62.47	61.7	96.79	91.91	95.64
Control-3	67.32	66.41	98.44	94.89	97.28
1/2 MIC-1	64.69	63.84	98.81	95.75	97.49
1/2 MIC-2	64.69	63.2	98.94	96.2	97.31
1/2 MIC-3	69.78	68.9	98.6	95.31	97.03
1 MIC-1	64.69	63.72	98.72	95.49	96.83
1 MIC-2	64.69	63.63	98.79	95.7	96.41
1 MIC-3	64.69	63.38	98.78	95.69	96.82

**Table 3:** Downregulated genes of flagellar assembly.

Gene ID	Gene Name	Description
N007_RS31875	*flhA*	Flagellar assembly
N007_RS31880	*fliR*	Flagellar type III secretion system protein FliR
N007_RS31885	*fliQ*	Flagellar biosynthesis protein FliQ
N007_RS31890	*fliP*	Flagellar assembly
N007_RS31910	*fliY*	Bacterial chemotaxis; flagellar assembly
N007_RS31915	*fliM*	Flagellar motor switch protein FliM
N007_RS31920	*fliG*	Flagellar motor switch protein FliG
N007_RS31930	*FliF*	Flagellar M-ring protein FliF
N007_RS31935	*flgC*	Flagellar basal body rod protein FlgC
N007_RS31985	*flgB*	Flagellar basal body rod protein FlgB
N007_RS33715	*fliS*	Flagellar export chaperone FliS
N007_RS33720	*fliD*	Flagellar hook assembly protein FlgD
N007_RS34120	*flgL*	Flagellar assembly

## Data Availability

The data used to support the findings of this study can be made available by the corresponding author upon request.
